# Weighted Relative Group Entropies and Associated Fisher Metrics

**DOI:** 10.3390/e24010120

**Published:** 2022-01-13

**Authors:** Iulia-Elena Hirica, Cristina-Liliana Pripoae, Gabriel-Teodor Pripoae, Vasile Preda

**Affiliations:** 1 Faculty of Mathematics and Computer Science, University of Bucharest, Academiei 14, 010014 Bucharest, Romania; ihirica@fmi.unibuc.ro (I.-E.H.); gpripoae@fmi.unibuc.ro (G.-T.P.); 2 Department of Applied Mathematics, Bucharest University of Economic Studies, Piata Romana 6, 010374 Bucharest, Romania; cristinapripoae@csie.ase.ro; 3 “Gheorghe Mihoc-Caius Iacob” Institute of Mathematical Statistics and Applied Mathematics of Romanian Academy, No.13 Calea 13 Septembrie, Sector 5, 050711 Bucharest, Romania; 4 “Costin C. Kiritescu” National Institute of Economic Research of Romanian Academy, No.13 Calea 13 Septembrie, Sector 5, 050711 Bucharest, Romania

**Keywords:** Fisher metric, group Fisher metric, weighted entropy, relative group entropy, α-weighted group entropy

## Abstract

A large family of new α-weighted group entropy functionals is defined and associated Fisher-like metrics are considered. All these notions are well-suited semi-Riemannian tools for the geometrization of entropy-related statistical models, where they may act as sensitive controlling invariants. The main result of the paper establishes a link between such a metric and a canonical one. A sufficient condition is found, in order that the two metrics be conformal (or homothetic). In particular, we recover a recent result, established for α=1 and for non-weighted relative group entropies. Our conformality condition is “universal”, in the sense that it does not depend on the group exponential.

## 1. Introduction

### 1.1. History

The inhabitants of the Universe of Uncertainty are probability distribution functions (PDFs). One can try to understand them through their entropy, a property which provides us a measure of disorder. Since its discovery, in the second part of the 19th Century, entropy was investigated by functional, algebraic or analytical techniques. The first geometric tools arose in the 1920s, through the work of Fisher ([1]), whose information matrix is the germ for what today is known as the Fisher metric. The notion was generalized by Rao ([2,3]) in the 1940’s, who put it in the appropriate context of Riemannian geometry. After a 30 years gap, Efron ([4]) and Amari ([5,6]) reopened the interest in differential geometric invariants associated to statistical models. Since the 1980s, the geometrization of the parameters space of the PDFs evolved into a field with rapidly growing expanse, including (among others) the new topics of statistical manifolds and of the dual connections ([7,8,9,10,11]; see in [12] for a recent review).

Our paper is a piece of Riemannian geometry intended for entropy study. We construct a new family of Fisher-like metrics, canonically associated to some entropy functionals, and we establish a sufficient condition in order for two of these metrics to be conformal. For describing the recipe, we must first sketch the story of its three main ingredients: the *weighting procedure* of an entropy functional, the *relative group entropy* and the *group Fisher metric* associated to it.

Around 1970, Belis and Guiasu ([13,14]) defined and axiomatized the concept of weighted entropy. They modified an entropy formula, by multiplying the integrand (which contained quantitative, objective and probabilistic information) with a “weighting function”, susceptible to model qualitative, subjective and non-stochastic utility data. The notion proved useful in various applications and gave rise to many papers. We quote here but a few: Barbu et al. [15], Batty [16], Das [17], Guiasu [18], Kayal [19], Kelbert et al. [20], Smieja [21], Suhov [22], and Tunnicliffe et al. [23], where the interested reader may find more details.

The notion of relative group entropy is more recent ([24]), but, as a nice coincidence, its algebraic roots are contemporary with the papers of Rao about the Fisher metric and distance, previously quoted. These algebraic foundations are the (Lie) formal groups, defined by Bochner [25] in 1946, as a unifying tool between Lie groups and Lie algebras (see also Hazewinkel [26]). Tempesta used the formal group laws, in order to define universality classes of entropies ([27,28], see also [29,30]). Starting with a formal group logarithm

$$G\left(t\right)=\sum_{i=0}^{\infty }c_{i}\frac{t^{i+1}}{i+1},\;\left(c_{i}\in Q\;,c_{0}=1\right),$$

he defined ([27]) group entropy functionals of the form

$$S_{G}\left(p\right):=\int_{X}p\left(x\right)G\left(lnp{\left(x\right)}^{-1}\right)dx,$$

where *p* is a PDF on *X*. As an application, the relative entropy (also known as the Kullback–Leibler divergence) was generalized ([27]) to the relative group entropy.

Gomez et al. used ([24]) the relative group entropies for defining group Fisher metrics, as “universal” extensions of the classical Fisher metrics arising from the classical Boltzmann-Gibbs entropy. Their main result states that the previous pairs of metrics are homothetic, and the constant of homothety depends on $G^{\prime }\left(0\right)$ and $G^{\prime \prime }\left(0\right)$.

### 1.2. Motivation

Our vision about entropy will be naive and formal, without entering into deep interpretation about the physical or informational meanings. Our goal is to filter the details and to see the nude invariants of geometric nature which might enlighten us to the general behavior of the various (and apparently too many) types of entropy avatars. For more details and hints about statistical interpretations, the reader is encouraged to look into the inspirational papers [24,27,31,32].

### 1.3. Contents of the Paper

In Section 2, we try to systematize and to unify different definitions for the entropy functions associated to PDFs. Two procedures of refinement are recalled: through “weighting” and through “powering” the PDFs. We shall need both in Section 4 and Section 5.

In Section 3, we review, in a creative manner, various methods to obtain semi-Riemannian metrics associated to families of entropy functions or functionals, as part of what we call “the geometrization problem for the entropy”. In particular, we have the Fisher metrics, the Hessian metrics and the group Fisher metrics. Generalizations include “mean” Hessian metrics, with the (“partial”) Hessian appearing under the integral sign.

Section 4 is the main part of the paper and is devoted to a generalization of the result of Gomez et al. [24]. The Theorem 1 provides a formula, linking a canonical weighted and powered Fisher-like metric to another one, which is, moreover, associated to a group exponential. We give a sufficient condition in order the two metrics be conformal or even homothetic. This conformality condition is, in some sense, “universal”, in that it does not depend on the group exponential.

Section 5 contains examples which illustrate the application of the general method from Section 4. We consider a normal PDF on the real line, with two parameters, with particular weighting functions and particular powers. As the compatibility relation is satisfied, it follows that, for *any* formal group exponential, we can construct a weighted Fisher metric, homothetic with the standard one. In addition to the construction of homothetic metrics in [24], our examples provide an additional control tool, through the weighting function. Moreover, our examples are well suited to be easily adapted and extended in more general frameworks.

### 1.4. Conventions

All the integrals are supposed to be correctly defined. Partial derivatives are supposed to commute with the integral. All the geometric objects are supposed to be differentiable, even if, in some cases, a weaker assumption would suffice (for example continuity or integrability).

## 2. Preliminaries

We review in a creative way some entropy-related notions, following mainly those in [14,27,28].

Consider $X\subset R^{m}$ the domain of a real valued random variable *x*. Let p=p(x) be a (differentiable) probability density function (PDF), with p(x)≥0 and $\int_{X}p\left(x\right)dx=1$. Let f:R×X→R be a differentiable *controlling* function.

**Definition** **1.** *The* generalized entropy *corresponding to the pair (p,f) is the real number $H=H^{\left(p,f\right)}$, where*

(1) $$H:=-\int_{X}f\left(p\left(x\right),x\right)dx\;.$$

*If the controlling function f depends on p and does not depend on x, we write f:R→R and we say the entropy*

(2) $$H=-\int_{X}f\left(p\left(x\right)\right)dx$$

*is classical.*

Using the convention in Section 1, we may write (1) and (2) in the equivalent *normalized* form

(3) $$H:=-\int_{X}p\left(x\right)h\left(p\left(x\right),x\right)dx,\;H=-\int_{X}p\left(x\right)h\left(p\left(x\right)\right)dx,$$

where f((p(x),x)=p(x)h((p(x),x) and f(p(x))=p(x)h(p(x)), respectively. Normalizing does not change the property of an entropy to be generalized or classical, respectively.

A slightly more general type of normalization is to consider some positive real number α and the entropy functions of the form

$$H^{\left(\alpha \right)}:=-\int_{X}p^{\alpha }\left(x\right)h\left(p\left(x\right),x\right)dx,\;H^{\left(\alpha \right)}=-\int_{X}p^{\alpha }\left(x\right)h\left(p\left(x\right)\right)dx.$$

This “powering” of the PDF acts as a control tool over the set of entropy functions and enables -sometimes- a useful refinement of the models. However, we must be aware of the fact that, with an appropriate notation, the previous entropy functions may be rewritten in the form (3).

**Example** **1.** *Almost all the known entropies used in statistical mechanics and information geometry are classical. We list but a few, from simple to more sophisticated ones.*

*(i) The functional f(p):=plog(p) gives the Boltzmann–Gibbs–Shannon (BGS) entropy.*

*(ii) For any fixed a∈R\{1}, $f\left(p\right):=p\;\frac{p^{a-1}-1}{a-1}$ provides a Tsallis entropy; when a→1 we recover the BGS entropy.*

*(iii) Considering one more PDF q, the functional $f\left(p\right):=p\;log\left(\frac{q}{p}\right)$ leads to the* relative entropy *(also known as the Kullback–Leibler divergence [24]) between them, which is the normalized classical entropy*

(4) $$D\left(p\|q\right):=\int_{X}p\left(x\right)log\left(\frac{p(x)}{q(x)}\right)dx.$$

*We accept (formally) that $0\cdot log(\frac{0}{q})=0$ and $p\cdot log(\frac{p}{0})=0$. More generally, we may start with k≥1 more PDFs $p_{1}$, …, $p_{k}$ and a differentiable function $F:R^{k+1}\to R$. Then, the functional $f\left(p\right):=F\left(p,p_{1},\ldots ,p_{k}\right)$ produces a relative entropy of p w.r.t. $p_{1}$,…, $p_{k}$, namely, the normalized classical entropy*

(5) $$D\left(p\|p_{1},\ldots ,p_{k}\right):=\int_{X}p\left(x\right)F\left(p\left(x\right),p_{1}\left(x\right),\ldots ,p_{k}\left(x\right)\right)dx.$$

*Moreover, this one may be viewed as a generalized entropy associated to the functional $p_{i}\to F\left(p,p_{1},\;\ldots ,p_{k}\right)$, for every $i=\bar{1,k}$.*

*(iv) Let G=G(t) be a formal group logarithm, which is a differentiable real valued function with some special algebraic properties, inspired from the formal series linking Lie groups to Lie algebras. (We refer to [24,27,28] for details about these functions). The* group entropy functional *associated to it is defined by ([24,27])*

(6) $$S_{G}\left(p\right):=\int_{X}p\left(x\right)G\left(lnp{\left(x\right)}^{-1}\right)dx,$$

*which is a normalized classical entropy of the form (3).*

*If q is another PDF, then the* relative group entropy *functional of p and q is defined ([24]) by*

(7) $$D_{G}\left(p\|q\right):=\int_{X}p\left(x\right)G\left(ln{\left(\frac{q(x)}{p(x)}\right)}^{-1}\right)dx,$$

*which is another normalized classical entropy of the form (3). Generalization for k PDFs leading to a relative group entropy of the form (5) is also possible, but we leave this step to the reader.*

**Remark** **1.** *Consider, moreover, an arbitrary differentiable function w:X→R. The* weighted generalized entropy *corresponding to the triple (p,f,w), associated to the generalized entropy (1), is another generalized entropy, given by*

$$H:=-\int_{X}w\left(x\right)f\left(p\left(x\right),x\right)dx.$$

*Similar weighted entropies may be defined starting from (2) and (3). For example, the weighted Tsallis entropy writes*

$$H=-\int_{X}w\left(x\right)p\left(x\right)\frac{({p(x))}^{a-1}-1}{a-1}dx.$$

*Weighting does not change the property of an entropy to be generalized or normalized, respectively. Instead, weighting may transform a classical entropy into a generalized one.*

## 3. Fisher Metrics for Generalized Entropy Functions

We review in a creative way the main notions related to the Fisher metric derived from a family of generalized entropies, associated to some parameterized PDFs, following mainly the work in [24].

Consider the case when the PDF *p* in Section 2 depends, moreover, on *n* real parameters $\theta^{1},\ldots ,\theta^{n}$, that is, $p:X\times R^{n}\to R$, p=p(x,θ), with $\theta :=(\theta^{1},\ldots ,\theta^{n})$. Let $f:R\times X\times R^{n}\to R$ be a differentiable *controlling* function, f=f(p,x,θ). The dependence on the parameter θ provides, via the relation (1), a generalized entropy function (loosely denoted also by) $H:R^{n}\to R$, given by

(8) $$H\left(\theta \right)=-\int_{X}f\left(p\left(x,\theta \right),x,\theta \right)dx.$$

Analogously, classical entropy functions and normalized ones arise naturally, from parameterizing with θ the relations (2) and (3), respectively.

**The geometrization problem for the entropy functions.** *Associate a relevant geometric structure to the function H (given by (8) or by any other avatar), whose invariants might provide information about the PDF p. Use f as a control tool in this process.*

**Remark** **2.** *The fist idea that comes to mind is to consider the (classical) Hessian tensor field associated to H, with coefficients*

(9) $${\left(Hess_{H}\right)}_{ij}\left(\theta \right):=-\int_{X}\frac{\partial^{2}f\left(p\left(x,\theta \right),x,\theta \right)}{\partial \theta^{i}\partial \theta^{j}}dx,\;i,j=\bar{1,n}.$$

*If $Hess_{H}$ is non-degenerate, it provides a semi-Riemannian metric (of constant signature) on $R^{n}$, called Hessian metric. Its geometry was subject to many papers (see, for example, in [33,34] and the references therein) and is useful in understanding the extremum points of H, as a semi-Riemannian optimization topic.*

*A simple example is the Euclidian metric $g_{ij}=\delta_{ij}$, arising as Hessian metric from Formula (9), by taking n=2, $\theta =(\theta^{1},\theta^{2})$ and $f\left(p\left(x,\theta \right),x,\theta \right):=-\frac{{\left(\theta^{1}\right)}^{2}+{\left(\theta^{2}\right)}^{2}}{2}p\left(x,\theta \right)$.*

**Remark** **3.** *On another hand, consider a controlling function $h:R\times R^{n}\to R$, with h=h(p,θ), and its associated normalized classical entropy function*

(10) $$H\left(\theta \right)=-\int_{X}p\left(x,\theta \right)h\left(p\left(x,\theta \right),\theta \right)dx.$$

*Define*

(11) $$g_{ij}\left(\theta \right):=-\int_{X}p\left(x,\theta \right)\frac{\partial^{2}h\left(p\left(x,\theta \right),\theta \right)}{\partial \theta^{i}\partial \theta^{j}}dx,\;i,j=\bar{1,n}.$$

*If the matrix ${\left(g_{ij}\right)}_{i,j=\bar{1,n}}$ is nowhere vanishing, we obtain the (semi-Riemannian) Fisher metric associated to H (or h) [24]. An example is included in Section 5, for a PDF of exponential type.*

*Denote $\tilde{h}\left(x,\theta \right):=h\left(p\left(x,\theta \right),\theta \right)$. Then,*

(12) $$g_{ij}\left(\theta \right)=-\int_{X}p\left(x,\theta \right)\frac{\partial^{2}\tilde{h}\left(x,\theta \right)}{\partial \theta^{i}\partial \theta^{j}}dx=-\int_{X}p\left(x,\theta \right){\left(Hess_{\tilde{h}}\right)}_{ij}\left(x,\theta \right)dx\;,\;i,j=\bar{1,n}.$$

*This formula does not define (in general) a Hessian metric. In fact, this might be called “mean” Hessian metric, as we do not derivate the whole integral, but only a factor of the integrand. (Here we consider the Hessian for $\tilde{h}$ as function depending on θ only.)*

*However, in some particular cases, the Fisher metrics may (eventually) borrow the appearance of a Hessian metric, but w.r.t. other appropriate function, as in the following case [34]. Consider the PDF of exponential type $p:X\times R^{n}\to R$, p=p(x,θ), with $\theta :=(\theta^{1},\ldots ,\theta^{n})$,*

$$p\left(x,\theta \right):=exp\{C\left(x\right)+\sum_{i=1}^{n}F_{i}\left(x\right)\theta^{i}-\nu \left(\theta \right)\},$$

*where C=C(x), $F_{1}=F_{1}\left(x\right)$,…, $F_{n}=F_{n}\left(x\right)$ and ν=ν(θ) are smooth functions. The associated Fischer metric on $R^{n}$ is $g=Hess_{\nu }$, a Hessian metric w.r.t. ν, which is not derived from an entropy function like in Formula (9).*

**Example** **2.** *In the case of the BCS-entropy, h=log∘p, $\tilde{h}\left(x,\theta \right)=log\left(p\left(x,\theta \right)\right)$ and we get the well-known classical Fisher metric*

$$g_{ij}\left(\theta \right)=-\int_{X}p\left(x,\theta \right){\left(Hess_{\tilde{h}}\right)}_{ij}\left(x,\theta \right)dx=\int_{X}p\left(x,\theta \right)\frac{\partial log(p(x,\theta ))}{\partial \theta^{i}}\frac{\partial log(p(x,\theta ))}{\partial \theta^{j}}dx.$$

*Its scalar curvature is interpreted as the average statistical uncertainty of a density matrix ([24]). This claim is quite natural, as the scalar curvature is obtained from the curvature tensor by taking the trace (a “mean”) two times successively, while the curvature tensor measures a “force” associated to the “matter” in the “Universe” $R^{n}$ driven by g.*

*Denote the entropy function $H\left(\theta \right)=-\int_{X}p\left(x,\theta \right)log\left(p\left(x,\theta \right)\right)dx$. We calculate*

$${\left(Hess_{H}\right)}_{ij}\left(\theta \right)=\int_{X}p\left(x,\theta \right){\left(Hess_{\tilde{h}}\right)}_{ij}\left(x,\theta \right)dx-\int_{X}{\left(Hess_{p}\right)}_{ij}\left(x,\theta \right)log\left(p\left(x,\theta \right)\right)dx=$$

$$=-g_{ij}\left(\theta \right)-\int_{X}\frac{\partial^{2}p}{\partial \theta^{i}\partial \theta^{i}}\left(x,\theta \right)log\left(p\left(x,\theta \right)\right)dx$$

*This formula expresses the Fischer metric g in terms of the Hessian of H and of the “mean” Hessian of p, “weighted” by $\tilde{h}$.*

**Remark** **4.** *(i) Consider now a classical entropy function $H:R^{n}\to R$, given by*

$$H\left(\theta \right)=-\int_{X}f\left(p\left(x,\theta \right)\right)dx$$

*and an associated weighted classical entropy function $H^{w}:R^{n}\to R$, given by*

$$H^{w}\left(\theta \right)=-\int_{X}w\left(x\right)f\left(p\left(x,\theta \right)\right)dx.$$

*Their Hessians have the components*

$${\hat{g}}_{ij}\left(\theta \right):=\frac{\partial^{2}H}{\partial \theta^{i}\partial \theta^{j}}\left(\theta \right)=-\int_{X}\frac{\partial^{2}f\left(p\left(x,\theta \right)\right)}{\partial \theta^{i}\partial \theta^{j}}dx$$

*and*

$${\left({\hat{g}}^{w}\right)}_{ij}\left(\theta \right):=\frac{\partial^{2}H^{w}}{\partial \theta^{i}\partial \theta^{j}}\left(\theta \right)=-\int_{X}w\left(x\right)\frac{\partial^{2}f\left(p\left(x,\theta \right)\right)}{\partial \theta^{i}\partial \theta^{j}}dx.$$

*In case of simultaneous non-degeneracy, the corresponding (semi-Riemannian) Hessian metrics $\hat{g}$ and ${\hat{g}}^{w}$ provide a (geo)metric tool for studying the impact of the weighting function w upon the entropy of the system.*

*(ii) Suppose the entropy function and the weighted entropy function are given in the normalized form*

$$H\left(\theta \right)=-\int_{X}p\left(x,\theta \right)h\left(p\left(x,\theta \right)\right)dx$$

*and*

$$H^{w}\left(\theta \right)=-\int_{X}w\left(x\right)p\left(x,\theta \right)h\left(p\left(x,\theta \right)\right)dx.$$

*Denote f(p(x,θ)):=p(x,θ)h(p(x,θ)). Then, we may calculate the components of the respective Hessians $\hat{g}$ and ${\hat{g}}^{w}$, as in (i). Eventually, in case of non-degeneracy, we get Hessian metrics and follow the strategy from (i).*

*For H and $H^{w}$, we may associate also the “mean” Hessian metrics g and $g^{w}$, as in (12). Therefore, we have the Fisher metrics on $R^{n}$, given by*

$$g_{ij}\left(\theta \right)=-\int_{X}p\left(x,\theta \right)\frac{\partial^{2}h\left(p\left(x,\theta \right)\right)}{\partial \theta^{i}\partial \theta^{j}}dx$$

*and*

$${\left(g^{w}\right)}_{ij}\left(\theta \right)=-\int_{X}w\left(x\right)p\left(x,\theta \right)\frac{\partial^{2}h\left(p\left(x,\theta \right)\right)}{\partial \theta^{i}\partial \theta^{j}}dx$$

*(iii) Similar Hessians or “mean” Hessians can be associated to relative entropy functions, as in Example 1,(iii), or to entropy group functionals, as in Example 1, (iv). If non-degenerate, they become semi-Riemannian Hessian metrics or Fisher metrics ([24,27,28]).*

*(iv) Analogous entropy functions and metrics arise when weighting by functions w=w(x,θ), instead of w=w(x). In fact, this is a more flexible weighting procedure, as it does not impose universal weighting constraints upon the whole family of PDFs.*

## 4. A Step Further: Passing from the Fisher Metric Group to a Weighted One

Consider a PDF *p* depending on *n* real parameters $\theta^{1},\ldots ,\theta^{n}$; that is, $p:X\times R^{n}\to R$, p=p(x,θ), with $\theta :=(\theta^{1},\ldots ,\theta^{n})$. Let *g* be the Fischer (semi)-Riemannian metric on $R^{n}$, whose coordinate functions are given by

$$g_{ij}\left(\theta \right)=\int_{X}p\left(x,\theta \right)\frac{\partial log(p(x,\theta ))}{\partial \theta^{i}}\frac{\partial log(p(x,\theta ))}{\partial \theta^{j}}dx.$$

Let G=G(t) be a formal group logarithm, as in Example 1, (iv). Consider $H:R^{n}\times R^{n}\to R$ the double parameterized group entropy functional associated to it ([24]), by

(13) $$H\left(\theta ,\tilde{\theta }\right):=\int_{X}p\left(x,\theta \right)\;G\left(ln\frac{p(x,\theta )}{p(x,\tilde{\theta })}\right)dx.$$

The Hessian tensor field $g_{G}$, with components

$${\left(g_{G}\right)}_{ij}\left(\tilde{\theta }\right):=\frac{\partial^{2}H\left(\theta ,\tilde{\theta }\right)}{\partial \theta^{i}\partial \theta^{j}}{|}_{\theta =\tilde{\theta }},$$

is called the group Fisher metric in [24]. The main result in [24] states that the metrics *g* and $g_{G}$ are homothetic, via the formula

(14) $$g_{G}=\left(G^{\prime }\left(0\right)+G^{\prime \prime }\left(0\right)\right)g.$$

From it follows the well-known correspondence between the main invariants of *g* and $g_{G}$, such as the Christoffel coefficients, the Riemann curvature coefficients and the scalar curvatures.

We prove now that this result remains true, in the more general assumption of α-weighted group entropy functionals.

**Theorem** **1.** *Let α be a positive real number. Let $w:X\times R^{n}\to R$ be a weighting function for (13), providing the double-parameterized weighted α-normalized group entropy functional*

(15) $$H^{w,\alpha }\left(\theta ,\tilde{\theta }\right):=\int_{X}w\left(x,\tilde{\theta }\right)p^{\alpha }\left(x,\theta \right)\;G\left(ln\frac{p(x,\theta )}{p(x,\tilde{\theta })}\right)dx.$$

*Consider the α-weighted group Fisher metric $g_{G}^{w,\alpha }$, with components*

$${\left(g_{G}^{w,\alpha }\right)}_{ij}\left(\tilde{\theta }\right):=\frac{\partial^{2}H^{w,\alpha }\left(\theta ,\tilde{\theta }\right)}{\partial \theta^{i}\partial \theta^{j}}{|}_{\theta =\tilde{\theta }}\;=\int_{X}w\left(x,\tilde{\theta }\right)\frac{\partial^{2}}{\partial \theta^{i}\partial \theta^{j}}\left\{p^{\alpha }\left(x,\theta \right)\;G\left(ln\frac{p(x,\theta )}{p(x,\tilde{\theta })}\right)\right\}{|}_{\theta =\tilde{\theta }}dx\;,$$

*and the α-weighted Fisher metric $g^{w,\alpha }$, with components*

$${\left(g^{w,\alpha }\right)}_{ij}\left(\theta \right):=\int_{X}w\left(x,\theta \right)p^{\alpha }\left(x,\theta \right)\frac{\partial log(p(x,\theta ))}{\partial \theta^{i}}\frac{\partial log(p(x,\theta ))}{\partial \theta^{j}}dx.$$

*Then, the metrics $g^{w,\alpha }$ and $g_{G}^{w,\alpha }$ are related, via the formula*

(16) $${\left(g_{G}^{w,\alpha }\right)}_{ij}\left(\theta \right)=\left\{\left(2\alpha -1\right)G^{\prime }\left(0\right)+G^{\prime \prime }\left(0\right)\right\}{\left(g^{w,\alpha }\right)}_{ij}\left(\theta \right)\;+G^{\prime }\left(0\right)\int_{X}w\left(x,\theta \right)p^{\alpha -1}\left(x,\theta \right)\;\frac{\partial^{2}p\left(x,\theta \right)}{\partial \theta^{i}\partial \theta^{j}}dx.$$

**Proof.**  We begin by deriving

$$\frac{\partial H^{w,\alpha }}{\partial \theta^{j}}\left(\theta ,\tilde{\theta }\right)=\int_{X}\alpha w\left(x,\tilde{\theta }\right)p^{\alpha -1}\left(x,\theta \right)\;\frac{\partial p(x,\theta )}{\partial \theta^{j}}\;G\left(ln\frac{p(x,\theta )}{p(x,\tilde{\theta })}\right)dx$$

$$\;+\int_{X}w\left(x,\tilde{\theta }\right)p^{\alpha -1}\left(x,\theta \right)\;\frac{\partial p(x,\theta )}{\partial \theta^{j}}\;G^{\prime }\left(ln\frac{p(x,\theta )}{p(x,\tilde{\theta })}\right)dx,$$

$$\frac{\partial^{2}H^{w,\alpha }}{\partial \theta^{i}\partial \theta^{j}}\left(\theta ,\tilde{\theta }\right)=\int_{X}\alpha \left(\alpha -1\right)w\left(x,\tilde{\theta }\right)p^{\alpha -2}\left(x,\theta \right)\;\frac{\partial p(x,\theta )}{\partial \theta^{i}}\;\frac{\partial p(x,\theta )}{\partial \theta^{j}}\;G\left(ln\frac{p(x,\theta )}{p(x,\tilde{\theta })}\right)dx$$

$$\;+\int_{X}\alpha w\left(x,\tilde{\theta }\right)p^{\alpha -1}\left(x,\theta \right)\;\frac{\partial^{2}p\left(x,\theta \right)}{\partial \theta^{i}\partial \theta^{j}}\;G\left(ln\frac{p(x,\theta )}{p(x,\tilde{\theta })}\right)dx$$

$$\;+\int_{X}\alpha w\left(x,\tilde{\theta }\right)p^{\alpha -2}\left(x,\theta \right)\;\frac{\partial p(x,\theta )}{\partial \theta^{i}}\;\frac{\partial p(x,\theta )}{\partial \theta^{j}}\;G^{\prime }\left(ln\frac{p(x,\theta )}{p(x,\tilde{\theta })}\right)dx$$

$$+\int_{X}\left(\alpha -1\right)w\left(x,\tilde{\theta }\right)p^{\alpha -2}\left(x,\theta \right)\;\frac{\partial p(x,\theta )}{\partial \theta^{i}}\;\frac{\partial p(x,\theta )}{\partial \theta^{j}}\;G^{\prime }\left(ln\frac{p(x,\theta )}{p(x,\tilde{\theta })}\right)dx$$

$$\;+\int_{X}w\left(x,\tilde{\theta }\right)p^{\alpha -1}\left(x,\theta \right)\;\frac{\partial^{2}p\left(x,\theta \right)}{\partial \theta^{i}\partial \theta^{j}}\;G^{\prime }\left(ln\frac{p(x,\theta )}{p(x,\tilde{\theta })}\right)dx$$

$$\;+\int_{X}w\left(x,\tilde{\theta }\right)p^{\alpha -2}\left(x,\theta \right)\;\frac{\partial p(x,\theta )}{\partial \theta^{i}}\;\frac{\partial p(x,\theta )}{\partial \theta^{j}}\;G^{\prime \prime }\left(ln\frac{p(x,\theta )}{p(x,\tilde{\theta })}\right)dx.$$

We assign $\theta :=\tilde{\theta }$ and use the properties: G(0)=0 and $\int_{X}p\left(x,\theta \right)dx=1$. We get

$${\left(g_{G}^{w,\alpha }\right)}_{ij}\left(\tilde{\theta }\right):=\left[\left(2\alpha -1\right)G^{\prime }\left(0\right)+G^{\prime \prime }\left(0\right)\right]\int_{X}w\left(x,\tilde{\theta }\right)p^{\alpha -2}\left(x,\tilde{\theta }\right)\;\frac{\partial p(x,\tilde{\theta })}{\partial \theta^{i}}\;\frac{\partial p(x,\tilde{\theta })}{\partial \theta^{j}}dx$$

$$+\;G^{\prime }\left(0\right)\int_{X}w\left(x,\tilde{\theta }\right)p^{\alpha -1}\left(x,\tilde{\theta }\right)\;\frac{\partial^{2}p\left(x,\tilde{\theta }\right)}{\partial \theta^{i}\partial \theta^{j}}dx$$

$$=\left[\left(2\alpha -1\right)G^{\prime }\left(0\right)+G^{\prime \prime }\left(0\right)\right]\int_{X}w\left(x,\tilde{\theta }\right)p^{\alpha }\left(x,\tilde{\theta }\right)\frac{\partial log(p\left(x,\tilde{\theta }\right))}{\partial \theta^{i}}\frac{\partial log(p\left(x,\tilde{\theta }\right))}{\partial \theta^{j}}dx$$

$$+\;G^{\prime }\left(0\right)\int_{X}w\left(x,\tilde{\theta }\right)p^{\alpha -1}\left(x,\tilde{\theta }\right)\;\frac{\partial^{2}p\left(x,\tilde{\theta }\right)}{\partial \theta^{i}\partial \theta^{j}}dx$$

$$=\left[\left(2\alpha -1\right)G^{\prime }\left(0\right)+G^{\prime \prime }\left(0\right)\right]{\left(g^{w,\alpha }\right)}_{ij}\left(\tilde{\theta }\right)+G^{\prime }\left(0\right)\int_{X}w\left(x,\tilde{\theta }\right)p^{\alpha -1}\left(x,\tilde{\theta }\right)\;\frac{\partial^{2}p\left(x,\tilde{\theta }\right)}{\partial \theta^{i}\partial \theta^{j}}dx,$$

which concludes the proof. □

**Corollary** **1.** *In the hypothesis and with the notations from Theorem 1, suppose there exists a function $\varphi :R^{n}\to R$, such that, for every $i,j=\bar{1,n}$,*

(17) $$\int_{X}w\left(x,\theta \right)p^{\alpha -1}\left(x,\theta \right)\;\frac{\partial^{2}p\left(x,\theta \right)}{\partial \theta^{i}\partial \theta^{j}}dx\;=\varphi \left(\theta \right)\int_{X}w\left(x,\theta \right)p^{\alpha -2}\left(x,\theta \right)\;\frac{\partial p(x,\theta )}{\partial \theta^{i}}\;\frac{\partial p(x,\theta )}{\partial \theta^{j}}dx.$$

*Then the metrics $g^{w,\alpha }$ and $g_{G}^{w,\alpha }$ are conformal, via the formula*

(18) $$g_{G}^{w,\alpha }=\left\{\left(2\alpha -1\right)G^{\prime }\left(0\right)+\varphi \;G^{\prime }\left(0\right)+G^{\prime \prime }\left(0\right)\right\}g^{w,\alpha }.$$

*If, moreover, φ is constant, then the metrics $g^{w,\alpha }$ and $g_{G}^{w,\alpha }$ are homothetic.*

**Remark** **5.** *(i) In [24], the notation $\theta_{0}$ is used, instead our notation $\tilde{\theta }$. We consider it more appropriate, for not creating the (possible wrong) impression of “constancy” of the variable.*

*(ii) We used the terminology from [24], when calling $g_{G}^{w,\alpha }$ a group Fisher entropy metric, but, in our opinion, it is but a Hessian-like metric, not Fisher-like (i.e., of a “mean” Hessian type, see Section 2).*

*(iii) Taking w(x)=1 and α=1 in Corollary 1, we get φ=0 and we recover the result from [24], outlined in the preamble of the section.*

*(iv) The relation (17) writes also*

(19) $$\int_{X}w\left(x,\theta \right)p^{\alpha -2}\left(x,\theta \right)\left\{p\left(x,\theta \right)\;\frac{\partial^{2}p\left(x,\theta \right)}{\partial \theta^{i}\partial \theta^{j}}-\varphi \left(\theta \right)\frac{\partial p(x,\theta )}{\partial \theta^{i}}\;\frac{\partial p(x,\theta )}{\partial \theta^{j}}\right\}dx=0.$$

*A strong sufficient condition for (19) to hold true is the Hessian matrix of p (w.r.t. to θ) is proportional with the matrix ${\left(\frac{\partial p(x,\theta )}{\partial \theta^{i}}\;\frac{\partial p(x,\theta )}{\partial \theta^{j}}\right)}_{i,j}$. Relation (19) is satisfied iff the respective proportionality holds as a* mean *, through the intermediate of the integral, and weighted by $wp^{\alpha -2}$.*

*(v) We may also understand Corollary 1 in the following way: let p be a parameterized PDF with an entropy function (13); if there exists a weighting function w, a positive integer α, and a function φ, such that (19) is satisfied, then the metrics $g_{G}^{w,\alpha }$ and $g^{w,\alpha }$ are conformal, via (18). Thus, the search for pairs of conformal metrics is controlled by a triple “tool box”. A nontrivial example will be given in the next section.*

*Moreover, we may conjecture that given a PDF p, there exist w, φ and α such that (19) holds. Preliminary calculations provide hints that this might be true, at least for PDFs “of exponential type”.*

*(vi) In (18), the conformal factor depends on α, only through the intermediary of the “speed” of G around 0, not (also) through the “acceleration” of G around 0.*

*(vii) The condition (19) does not depend on G. In this sense, it is an “universal” condition, and we suspect it may hidden some (remarkable?) unraveled family of PDFs.*

*(viii) We introduce a new function ψ, related to the conformal factor in (18), by*

$$e^{2\psi }:=\left(2\alpha -1\right)G^{\prime }\left(0\right)+\varphi \;G^{\prime }\left(0\right)+G^{\prime \prime }\left(0\right).$$

*(We suppose the right side positive.) Denote $\rho_{G}^{w,\alpha }$ and $\rho^{w,\alpha }$ the scalar curvatures of $g_{G}^{w,\alpha }$ and $g^{w,\alpha }$, respectively. Then,*

$$e^{2\psi }\rho_{G}^{w,\alpha }=\rho^{w,\alpha }-2\left(n-1\right)\Delta \psi -\left(n-2\right)\left(n-1\right){\left(d\psi \right)}^{2}.$$

*Scenario: suppose p, w, α are given, such that the conformality condition (19) holds. Calculate the scalar curvature $\rho^{w,\alpha }$ (together with all the invariants associated to the metric $g^{w,\alpha }$). Then, the previous formula allows a conformal variation of $\rho^{w,\alpha }$, controlled by the set of formal group logarithms G, which is more flexible than the homothetic variation in [24]. Homothetic transformations of the metrics are quite rigid, as they preserve the qualitative behavior of the main invariants semi-Riemannian (distance, geodesics, curvature). By contrast, conformal non-homothetic transformations produce significant geometric changes, such as passing from a plane to a sphere.*

## 5. Examples

In what follows, consider X=R and the normal PDF p:R×R×(0,∞)→R,

(20) $$p\left(x;\theta^{1},\theta^{2}\right)=\frac{1}{\sqrt{2\pi }\theta^{2}}e^{-\frac{{\left(x-\theta^{1}\right)}^{2}}{2{\left(\theta^{2}\right)}^{2}}}.$$

Denote by $p_{1}$, $p_{2}$, $p_{11}$, $p_{12}$, $p_{22}$ the partial derivatives of *p*, of order one and two, w.r.t. $\theta^{1}$ and $\theta^{2}$. We calculate, successively,

$$p_{1}=\frac{x-\theta^{1}}{{\left(\theta^{2}\right)}^{2}}\;p,\;p_{2}=\left\{\frac{{\left(x-\theta^{1}\right)}^{2}}{{\left(\theta^{2}\right)}^{3}}-\frac{1}{\theta^{2}}\right\}\;p,$$

$$p_{11}=\left\{\frac{{\left(x-\theta^{1}\right)}^{2}}{{\left(\theta^{2}\right)}^{4}}-\frac{1}{{\left(\theta^{2}\right)}^{2}}\right\}\;p,\;p_{12}=\left\{\frac{{\left(x-\theta^{1}\right)}^{3}}{{\left(\theta^{2}\right)}^{5}}-\frac{3(x-\theta^{1})}{{\left(\theta^{2}\right)}^{3}}\right\}\;p,$$

$$p_{22}=\left\{\frac{{\left(x-\theta^{1}\right)}^{4}}{{\left(\theta^{2}\right)}^{6}}-\frac{5{\left(x-\theta^{1}\right)}^{2}}{{\left(\theta^{2}\right)}^{4}}+\frac{2}{{\left(\theta^{2}\right)}^{2}}\right\}\;p.$$

The classical Fisher metric *g* has the components $g_{11}={\left(\theta^{2}\right)}^{-2}$, $g_{12}=g_{21}=0$ and $g_{22}=2{\left(\theta^{2}\right)}^{-2}$ (see, for example, [10]).

Let α be a positive real number and consider the *particular* weighting function $w\left(x,\theta \right):={\left(x-\theta^{1}\right)}^{2}$. We calculate the coefficients of the α-weighted Fisher metric $g^{w,\alpha }$:

$$g_{11}^{w,\alpha }=3{\left(\sqrt{2\pi }\theta^{2}\right)}^{1-\alpha }\alpha^{-\frac{5}{2}},\;g_{12}^{w,\alpha }=0,\;g_{22}^{w,\alpha }=\left(\alpha^{2}-6\alpha +15\right){\left(\sqrt{2\pi }\theta^{2}\right)}^{1-\alpha }\alpha^{-\frac{7}{2}}.$$

We prove now that, in the particular case of $\alpha =\frac{3\;+\;\sqrt{57}}{2}$, with the function $\varphi :=1-\frac{\alpha }{3}=\frac{3\;-\;\sqrt{57}}{6}$, the relation (19) is identically satisfied. We evaluate the left side integral in (19), in a case-by-case calculation.

(i) The case i=1,j=2:

$$\int_{-\infty }^{\infty }{\left(x-\theta^{1}\right)}^{2}p^{\alpha }\left(x,\theta \right)\left\{\frac{{\left(x-\theta^{1}\right)}^{3}-3\left(x-\theta^{1}\right){\left(\theta^{2}\right)}^{2}}{{\left(\theta^{2}\right)}^{5}}-\varphi \frac{{\left(x-\theta^{1}\right)}^{3}-\left(x-\theta^{1}\right){\left(\theta^{2}\right)}^{2}}{{\left(\theta^{2}\right)}^{5}}\right\}dx=0\;,$$

as $p^{\alpha }$ is an even function and all the binomial integrands are odd functions, w.r.t. $\left(x-\theta^{1}\right)$. (We see that here we did not use the particular form of φ, so the result is valid for any positive α and any function φ=φ(θ).)

(ii) The case i=1,j=1:

$$\int_{-\infty }^{\infty }{\left(x-\theta^{1}\right)}^{2}p^{\alpha }\left(x,\theta \right)\left\{\frac{{\left(x-\theta^{1}\right)}^{2}-{\left(\theta^{2}\right)}^{2}}{{\left(\theta^{2}\right)}^{4}}-\left(1-\frac{\alpha }{3}\right)\frac{{\left(x-\theta^{1}\right)}^{2}}{{\left(\theta^{2}\right)}^{4}}\right\}dx=0\;.$$

(We see that here we did not use the particular form of α, so the result is valid for any positive α and for the function $\varphi =1-\frac{\alpha }{3}$.)

(iii) The case i=2,j=2:

$$\int_{-\infty }^{\infty }{\left(x-\theta^{1}\right)}^{2}p^{\alpha }\left(x,\theta \right)\{\frac{{\left(x-\theta^{1}\right)}^{4}-5{\left(x-\theta^{1}\right)}^{2}{\left(\theta^{2}\right)}^{2}+2{\left(\theta^{2}\right)}^{4}}{{\left(\theta^{2}\right)}^{6}}-$$

$$-\left(1-\frac{\alpha }{3}\right)\frac{{\left(x-\theta^{1}\right)}^{4}-2{\left(x-\theta^{1}\right)}^{2}{\left(\theta^{2}\right)}^{2}+{\left(\theta^{2}\right)}^{4}}{{\left(\theta^{2}\right)}^{6}}\}dx=k\left(\alpha^{2}-3\alpha -12\right)=0\;,$$

where *k* is a nowhere vanishing function, depending on α and $\theta^{2}$. (We see that here we used both the particular form of α and of the function φ.)

Let *G* be *any* formal group logarithm. As the relation (19) is verified for a *constant* function φ, it follows that the α-weighted group Fisher metric $g_{G}^{w,\alpha }$ is homothetic to $g^{w,\alpha }$. Using Formula (18), we obtain

(21) $$g_{G}^{w,\alpha }=\{\frac{5(3+\sqrt{57})}{6}\;G^{\prime }\left(0\right)+G^{\prime \prime }\left(0\right)\}g^{w,\alpha }.$$

**Remark** **6.** *Similar examples may be obtained: for $w\left(x,\theta \right):={\left(x-\theta^{1}\right)}^{a}$, with even a; for a polynomial function w with variable $\left(x-\theta^{1}\right)$ (the odd powers are irrelevant); for $w\left(x\right):=x^{a}$ (or a polynomial function in x), with a significant increase of the complexity of the calculations.*

**Remark** **7.** *Consider the PDF (20), a positive real number α and a weighting function $w\left(x,\theta \right)=l\left(\frac{{\left(x-\theta^{1}\right)}^{2}}{2{\left(\theta^{2}\right)}^{2}}\right)$, where l:R→R is an arbitrary continuous function (satisfying, eventually, some additional specific hypothesis, in order the following integrals exist). The cases i=1,j=1 and i=1,j=2 of the relation (19) are identically satisfied, for the constant function*

$$\varphi =1-\frac{\alpha }{2}\;\frac{\int_{0}^{\infty }l\left(\frac{t}{\alpha }\right)t^{-\frac{1}{2}}e^{-t}dt}{\int_{0}^{\infty }l\left(\frac{t}{\alpha }\right)t^{\frac{1}{2}}e^{-t}dt}.$$

*First, let us remark that this family of examples cannot lead to conformal non-homothetic transformations of the metric, via the relation (18). Second, we must impose that condition (19) holds, for i=2,j=2 also, and this provides a strong additional constraint on φ and α.*

*Unfortunately, the existence of a suitable value of the power α is not guaranteed. For example, in the particular case of $l\left(u\right):=1+e^{-u}$, we get*

$$\varphi =1-\frac{\alpha (\alpha +1)}{2\alpha +1-\sqrt{\alpha (\alpha +1)}}.$$

*Then, from the case for i=2,j=2 in (19), it follows that α is non-real or non-positive.*

**Example** **3.** *We consider now a particular case of the previous example. Let p=p(x,θ) be the two-variate PDF from (20); the weighting function $w\left(x,\theta \right)={\left(x-\theta^{1}\right)}^{2}$; $\alpha =\frac{3\;+\;\sqrt{57}}{2}$.*

*(i) Let G be the Thallis group logarithm, given [24] by*

$$G\left(t\right)=\frac{e^{(1-q)t}-1}{1-q},\;q\in R\;,\;q\neq 1.$$

*When q→1, we get G(t)→t, i.e. the BGS group logarithm. By abuse, from now on, we shall suppose q∈R.*

*Denote*

$$A:=3{\sqrt{2\pi }}^{-\frac{1+\sqrt{57}}{2}}{\left(\frac{3+\sqrt{57}}{2}\right)}^{-\frac{5}{2}},\;B:={\sqrt{2\pi }}^{-\frac{1+\sqrt{57}}{2}}\frac{45-3\sqrt{57}}{2}{\left(\frac{3+\sqrt{57}}{2}\right)}^{-\frac{7}{2}}.$$

*The α-weighted Fisher metric writes*

$$\left(g_{ij}^{w,\alpha }\right)=\left(\begin{array}{cc} A{\left(\theta^{2}\right)}^{1-\alpha } & 0\\ 0 & B{\left(\theta^{2}\right)}^{1-\alpha } \end{array}\right).$$

*Its Christoffel coefficients are*

$$\left(\Gamma_{ij}^{1}\right)=\left(\begin{array}{cc} 0 & \frac{1-\alpha }{2\theta^{2}}\\ \frac{1-\alpha }{2\theta^{2}} & 0 \end{array}\right),\;\left(\Gamma_{ij}^{2}\right)=\left(\begin{array}{cc} -\frac{A}{B}\frac{1-\alpha }{2\theta^{2}} & 0\\ 0 & \frac{1-\alpha }{2\theta^{2}} \end{array}\right).$$

*We calculate successively the Riemann–Christoffel coefficients*

$$R_{212}^{1}=\frac{\partial \Gamma_{22}^{1}}{\partial \theta^{1}}-\frac{\partial \Gamma_{21}^{1}}{\partial \theta^{2}}+\Gamma_{11}^{1}\Gamma_{22}^{1}+\Gamma_{21}^{1}\Gamma_{22}^{2}-\Gamma_{12}^{1}\Gamma_{21}^{1}-\Gamma_{22}^{1}\Gamma_{21}^{2}=\frac{1-\alpha }{2}\frac{1}{{\left(\theta^{2}\right)}^{2}}.$$

$$R_{1212}=g_{11}R_{212}^{1}=A{\left(\theta^{2}\right)}^{1-\alpha }\frac{1-\alpha }{2}\frac{1}{{\left(\theta^{2}\right)}^{2}}=\frac{A(1-\alpha )}{2}{\left(\theta^{2}\right)}^{-\alpha -1}.$$

*and the scalar curvature $\rho =\rho (\theta^{2})$,*

$$\rho =\frac{2R_{1212}}{\det(g_{ij})}=\frac{2\frac{A(1-\alpha )}{2}{\left(\theta^{2}\right)}^{-1-\alpha }}{AB{\left(\theta^{2}\right)}^{2-2\alpha }}=\frac{1-\alpha }{B}{\left(\theta^{2}\right)}^{\alpha -3}.$$

*We have $G^{\prime }\left(0\right)=1$ and $G^{\prime \prime }\left(0\right)=1-q$. From the relation (21) we calculate the scalar curvature of the associated α-weighted group Fisher metric $g_{G}^{w,\alpha }$:*

$$\rho_{G}\left(\theta^{2},q\right)={\left(\frac{21+5\sqrt{57}}{6}-q\right)}^{-1}\cdot \frac{1-\alpha }{B}{\left(\theta^{2}\right)}^{\alpha -3}.$$

*We restrain the study for $q<\frac{21\;+\;5\sqrt{57}}{6}$.*

**Figure 1:**
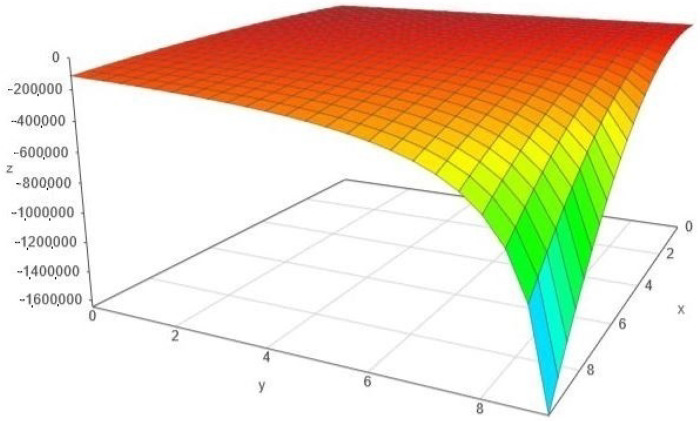
The variation of $\rho_{G}$, with the notation $x:=\theta^{2}$ and y:=q.

*As already pointed out in Example 2, the scalar curvature measures the average statistical uncertainty. In this case, both scalar curvature functions ρ and $\rho_{G}$ take strictly negative values, so the uncertainty is proportional to their module. The variation of $\rho_{G}$ is depicted in Figure 1.*

*(ii) If we replace the Tsallis group logarithm with the Kaniakidis group logarithm [24]*

$$G\left(t\right)=\frac{sinh(1-q)t}{1-q},\;q\in R\;,\;q\neq 1,$$

*the scalar curvatures ρ and $\rho_{G}$ differ by a constant only. The reason is that $G^{\prime }\left(0\right)=1$ and $G^{\prime \prime }\left(0\right)=0$, and $\rho_{G}$ gets no new variables from (21).*

*(iii) Instead, replacing the Tsallis group logarithm with the Abe group logarithm [24]*

$$G\left(t\right)=\frac{e^{at}-e^{bt}}{a-b},\;a,b\in R\;,\;a\neq b,$$

*leads to a behavior of $\rho_{G}$ versus ρ similar to (i), because $G^{\prime }\left(0\right)=1$ and $G^{\prime \prime }\left(0\right)=a+b$. In this case, $\rho_{G}$ depends on a, b and $\theta^{2}$.*

**Remark** **8.** *Let p=p(x,θ) be the two-variate PDF from (20); the weighting function $w\left(x,\theta \right)={\left(x-\theta^{1}\right)}^{2}$; α an arbitrary positive real number; G an arbitrary group logarithm. In general, the hypothesis of the Corollary 1 is not fulfilled anymore. This implies that the metrics $g^{w,\alpha }$ and $g_{G}^{w,\alpha }$ might not be conformal, and all we deduced previously for their scalar curvatures did not remain true, in general.*

*However, in this case we can study what can be derived, directly from Theorem 1, via the relation (16). A tedious calculation produces the following formulas:*

$${\left(g_{G}^{w,\alpha }\right)}_{11}=C\cdot {\left(\theta^{2}\right)}^{1-\alpha },\;{\left(g_{G}^{w,\alpha }\right)}_{12}=0,\;{\left(g_{G}^{w,\alpha }\right)}_{22}=D\cdot {\left(\theta^{2}\right)}^{1-\alpha },$$

*where we denoted*

$$C=\left[5\alpha \;G^{\prime }\left(0\right)+3G^{\prime \prime }\left(0\right)\right]\cdot {\sqrt{2\pi }}^{1-\alpha }\cdot \alpha^{-\frac{5}{2}}$$

$$D=\left[\left(2\alpha^{3}-11\alpha^{2}+21\alpha \right)\cdot G^{\prime }\left(0\right)+\left(\alpha^{2}-6\alpha +15\right)\cdot G^{\prime \prime }\left(0\right)\right]\cdot {\sqrt{2\pi }}^{1-\alpha }\cdot \alpha^{-\frac{7}{2}}$$

*The scalar curvature writes*

$$\rho_{G}=\frac{1-\alpha }{D}{\left(\theta^{2}\right)}^{\alpha -3}.$$

*Until now, G was arbitrary. Let us particularize it: consider G(t)=t, i.e., the BGS group logarithm. The scalar curvature of the corresponding α-weighted relative group metric is*

$$\rho_{G}=\frac{1-\alpha }{2\alpha^{3}-11\alpha^{2}+21\alpha }\cdot {\sqrt{2\pi }}^{\alpha -1}\cdot \alpha^{\frac{7}{2}}\cdot {\left(\theta^{2}\right)}^{\alpha -3}.$$

**Figure 2:**
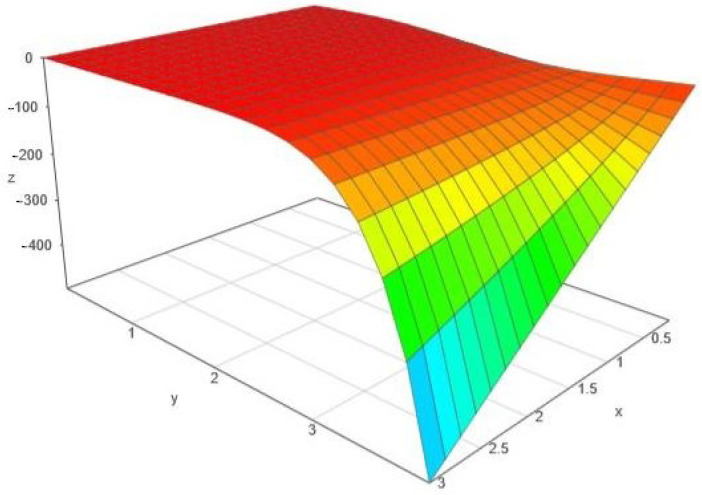
The variation of $\rho_{G}$, with the notation $x:=\theta^{2}$ and y:=α>0.

*Its “macroscopic” variation with respect to $\theta^{2}$ and α can be seen in Figure 2.*

**Figure 3:**
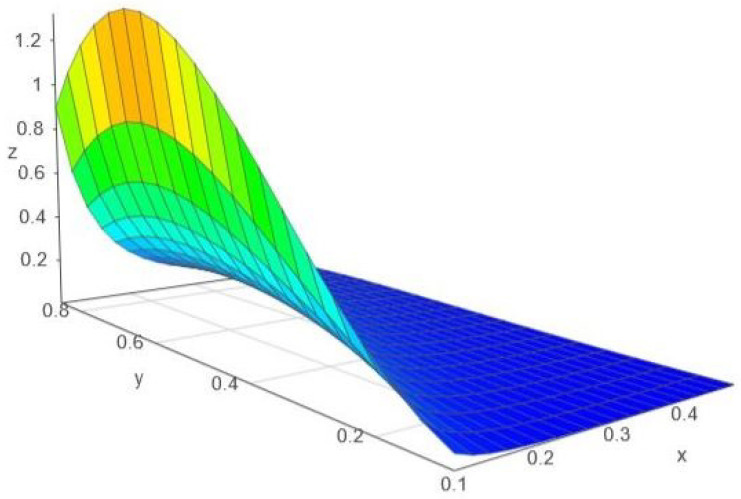
The variation of $\rho_{G}$, with the notation $x:=\theta^{2}$ and y:=α∈(0,1).

*We remark the special case α=1, when the metrics $g^{w,1}$ and $g_{G}^{w,1}$ become locally Euclidean and all the related curvature invariants (including the scalar curvature) vanish. For α>1, both scalar curvatures are negative and this behavior is clearly visible in Figure 1. For α∈(0,1), both scalar curvatures are positive; to see it, we must take a magnified detail, as in Figure 3.*

*Similar calculations can be made for other group logarithms also, following the same line. For the Tsallis group logarithm, the scalar curvature will depend also on the third variable q; for the Abe group logarithm, the scalar curvature will depend also on two more variables, namely, a and b.*

## 6. Discussion

The main motivation of our research was the discovery of new metrics, able to geometrize properties of statistical phenomena. We succeeded a double-folded generalization of the construction from in [24], extending the “homothetization” of the group Fisher metric to a “conformalization” of the α-weighted ones. These are semi-Riemannian metrics associated to entropy functions depending on arbitrary weighting functions and to powers of the PDFs. However, the generalization has a price: the existence of the conformal transformation requires a compatibility relation between the weighting functions, the powers of the PDFs and the PDFs (the relation (19)). We conjectured that, for a given PDF, there exists a weighting function and a power of the PDF, such that the relation (19) holds. We speculate that the elegance of (19) suggests a deeper property, whose hidden meaning remains to be revealed.

From now on, several directions of study open, hierarchically ordered upon the complexity/difficulty of the calculations involved:

(i) the constructions of new examples, by appropriate α-weighting the PDF from Section 5, as suggested in the Remark 6.

(ii) the constructions of new examples, similar to those in Section 5, for other remarkable PDFs on the real line, depending on two parameters (as the lognormal, the Gamma and the Beta distributions, following the classical approach from [10] ).

(iii) passing from m=1 and n=2 (as in (i) and (ii)) to arbitrary integers *m* and *n*, and appropriate α-weighting of *classical examples* of PDFs.

(iv) deepening the differential geometric study of the previous new metrics, and finding new geometric invariants for the modeling and the control of statistical phenomena. For example, the study of the distance related topics (see [35]), of the geodesics (see [36,37,38]) and of the various types of curvature tensor fields (see [10,39,40,41,42] for samples of the needed geometric tools).

(v) finding conformal, non-homothetic examples for which relation (19) holds. (These examples may also provide hints and support for our previously described conjecture.) Beyond the dreaded complications of the formalism, await the rewards of the much richer conformal geometry.

## Data Availability

Not applicable.
